# Effects of Freeze–Thaw Pretreatment and Cold-Press Dewatering on the Downstream Energy Demand of Hot-Air Drying of Whole Tomato By-Product Powder Production

**DOI:** 10.3390/foods15162791

**Published:** 2026-08-09

**Authors:** Adriana Lordi, Amalia Conte, Matteo Alessandro Del Nobile

**Affiliations:** 1 Department of Social Sciences, University of Foggia, Via Da Zara, 71122 Foggia, Italy; adriana.lordi@unifg.it; 2 Department of Humanistic Studies, Letters, Cultural Heritage, Educational Sciences, University of Foggia, Via Arpi, 71121 Foggia, Italy; 3 Department of Economics, Management and Territory, University of Foggia, Via A. Da Zara, 71122 Foggia, Italy; matteo.delnobile@unifg.it

**Keywords:** tomato, by-product, dehydration, freeze-thaw, cold-press extraction, energy demand, saving, sustainability

## Abstract

The valorization of vegetable processing by-products is increasingly recognized as a key strategy for improving sustainability and supporting circular economy principles. This study evaluated the effects of freeze–thaw pretreatment and cold-press dewatering prior to dehydration on the energy demand of whole tomato by-product processing. Tomato by-products were divided into different batches and subjected to freezing and thawing and/or to cold-press dewatering before dehydration at 60 °C. Only the cold-pressed samples, with or without freeze–thaw pretreatment, were also evaluated at 50 and 70 °C. A mathematical model previously reported in the literature was applied to estimate water removal during dehydration and quantify the energy demand associated with cold-press extraction, drying, and milling operations, enabling comparison among the investigated processing strategies. The results showed that the inclusion of a cold-press dewatering step decreased the energy demand, as the energy required for dewatering was lower than that needed to evaporate the same amount of water during drying. Freeze–thaw pretreatment further reduced the amount of free water to be removed in the subsequent dehydration stage. Although drying temperature had a limited effect on total energy demand, a slight decrease was calculated when increasing the temperature from 50 to 70 °C. These findings demonstrate the potential of combining freeze–thaw and cold-press dewatering as pretreatments for reducing energy demand throughout tomato by-product processing.

## 1. Introduction

The valorisation of agri-food by-products represents a crucial challenge for promoting sustainability and circular economy in the food sector [1]. Globally, approximately 1.3 billion tons of food is lost or wasted annually, with a significant fraction originating from agricultural production and industrial processing. This massive amount of waste not only represents a loss of valuable resources, but also imposes a substantial environmental and economic burden, with an estimated impact of about USD 1 trillion per year on the global economy [2]. Focusing on specific sectors, the tomato (*Solanum lycopersicum* L.) is one of the major contributors to this challenge, generating hundreds of thousands of tons of by-products rich in high-value components, especially lycopene, dietary fibre, and polyphenols [3,4]. Among the most abundant tomato by-products, whole tomato, unsuitable for primary processing due to failure to meet regulatory requirements concerning aesthetic defects, size, mechanical damage, or colour, holds high nutritional and functional potential [5]. However, its high initial moisture content (typically over 90% by mass) limits its direct application and complicates its long-term preservation [6]. The most common application for valorisation is the transformation of this by-product into stable powder or extracts for use as functional additives or food ingredients [7]. The conversion into a stable product is hampered by two main limitations, both linked to the high water content. The high water activity (aw) of fresh tomato is the primary factor exposing the product to rapid microbial deterioration and consequent loss of quality and safety [8]; furthermore, conventional processes for moisture reduction, such as thermal dehydration and compound extraction (solvent or thermal extraction), are energetically expensive and potentially damaging to heat-sensitive bioactive compounds, such as lycopene [9]. Increasing attention has been devoted to process intensification strategies aimed at reducing the amount of water that must be removed by evaporation during drying [10,11]. These strategies can be broadly classified into two categories: mechanical dewatering and drying pretreatments. Mechanical dewatering, including pressing, screw pressing, filtration, and centrifugation, removes water without a liquid-to-vapour phase transition, making it considerably less energy-intensive than thermal drying [12]. However, the efficiency of mechanical dewatering depends strongly on the structural and physicochemical characteristics of the processed biomass, requiring evaluation on different vegetable matrices [13]. Drying pretreatments, such as ultrasound, microwave, infrared, and pulsed electric fields, improve heat and mass transfer by modifying plant tissue structure. Although these technologies can shorten drying time and enhance process efficiency, they generally do not reduce the amount of water that must ultimately be evaporated. Consequently, the latent heat of water evaporation remains the major contributor to total energy consumption [14].

To address these limitations, the present study introduces a comparative evaluation of two unconventional pre-treatment strategies, aimed at drastically reducing the initial moisture before final drying. The first approach exploits the effect of freezing followed by thawing. Freezing causes the formation of ice crystals that perforate cell membranes and damage the microstructure of the plant tissue. During the thawing phase, this cellular damage results in the release of a significant amount of cytoplasmic water in the form of drip loss [15]. The thawed waste exhibits a lower moisture content compared to the fresh product, reducing the amount of water that must be actively removed through drying; furthermore, cell rupture can facilitate the subsequent removal of residual water during drying, lowering the internal resistance to mass transfer [16]. The second approach involves using a cold pressing technique as a pre-treatment step to separate the whole tomato into juice and solid residue. This technique has not yet been fully investigated in the specific context of by-product processing for industrial dehydration and therefore it represents a novel approach. The benefits derived are manifold: firstly, mechanical separation removes most of the aqueous juice, leading the solid residue to a lower moisture content; therefore, starting from a low-moisture solid fraction (and therefore potentially low aw) mitigates the risks of microbial growth during drying; moreover, being a cold and short-duration process, it optimally preserves thermosensitive compounds in the solid residue intended for powder [17]. In addition, this novel approach offers the additional opportunity to recover a liquid fraction naturally enriched in water-soluble bioactive compounds, thereby improving both the energy demand and the overall valorisation potential of the biomass within a circular bioeconomy framework.

Based on this rationale, the present research aimed to compare pretreatment strategies coupled to thermal dehydration, with respect to their energy demand during downstream processing of whole tomato by-products. To this aim, the performance of different dehydration protocols was compared by calculating the energy consumption.

## 2. Materials and Methods

### 2.1. Raw Materials

The whole tomato by-products were supplied by the same processing company (RossoGargano srl, Foggia, Italy) and consisted of a mixture of three commercial processing cultivars (Datterino, Ciliegino and San Marzano). The material was discarded before industrial processing because it did not meet commercial quality requirements, mainly due to non-conforming size and/or external colour, while remaining suitable for processing. The three cultivars were intentionally pooled to reproduce the heterogeneous composition of tomato by-products typically generated under industrial processing conditions. Since the objective of this study was to evaluate the effect of the proposed processing strategies on energy consumption associated specifically with cold-press extraction–dehydration–grinding, rather than the influence of cultivar or fruit maturity, these variables were not considered as experimental factors. Given the high moisture content of this by-product (approximately 95%), to prevent degradation and contamination, for each batch collected, a portion was immediately stored at −18 °C and a portion was temporarily refrigerated at 4 °C, to be used the day after. For freezing, a conventional domestic chest freezer operating at −18 °C for 48 h was used. Subsequently, the samples were thawed under refrigerated conditions at 4 °C until complete thawing before undergoing the cold-press dewatering process.

### 2.2. Whole Tomato By-Product Cold-Press Dewatering

Cold-press dewatering was performed using a low-speed screw juicer (Estraggo PRO, Siqur Salute Srl, Vigonza (PD), Italy). Whole tomato by-products were processed at room temperature by continuous feeding into the juicer hopper. The screw rotated at approximately 43 rpm (manufacturer specification), gently crushing and pressing the material against a fine stainless-steel filter, thereby separating it into a liquid juice fraction and a fibrous solid residue. The low rotational speed minimized frictional heat generation and limited oxidation during processing. No external heating was applied, and the temperature of the material was not monitored, although no perceptible temperature increase was observed during extraction. The residence time of the material within the extractor was not controlled because the process was operated continuously. The resulting solid residue exhibited a moisture content of approximately 85% and was subsequently subjected to convective drying. The extraction yielded about 77% (*w*/*w*) juice and 15% (*w*/*w*) solid residue from frozen by-products and about 80% (*w*/*w*) juice and 20% (*w*/*w*) solid residue from fresh by-products.

### 2.3. Tomato By-Product Dehydration and Grinding

The hot air-drying process of both whole tomato by-product and solid residue obtained by cold-press extraction was carried out using a conventional dryer (PF-SICCO80PRO, SICCO TECH, Campobasso, Italy) with forced convection at atmospheric pressure, testing different dehydration temperatures (50, 60 and 70 °C) and setting the relative humidity at 5%. The drying kinetics, calculated as in the study conducted by Lordi et al. [18], allowed us to estimate the time (24 h) necessary to dehydrate these by-products. Approximately 6 kg of fresh material was processed in each drying batch. The samples were uniformly distributed over multiple stainless-steel mesh trays to form a thin and homogeneous layer, minimizing particle overlap and promoting uniform air exposure throughout the drying process. The dryer has an internal chamber volume of 0.6 m^3^ and uses a forced-air circulation system to ensure uniform heat distribution inside the drying chamber. Air velocity and product temperature were not directly monitored during the experiments but were governed by the operating conditions of the equipment. Following drying, the material was allowed to cool to room temperature before milling. The resulting dehydrated powders were ground in a laboratory grinder (Peanut Machine, KMEC Engineering, Kate Road, Anqiu, China), obtaining a very fine powder (<500 μm), which was stored in plastic bags at 4 °C, protected from light for further applications.

### 2.4. By-Product Water Content

The by-product water content was evaluated according to the following equation:

(1) $$C\left(t\right)\;=\;\frac{W\left(t\right)-\;W_{F}}{W_{F}}\cdot 100$$

where $C\left(t\right)$ is the sample water content at time t expressed as $\left[\frac{g\;water}{100\;g\;dry\;matter}\right]$; $W\left(t\right)$ is the weight of sample at time t expressed as $\left[g\right]$; and $W_{F}$ is the weight of the sample after it has been kept at 130 °C until all the water was desorbed. It was expressed as $\left[g\right]$.

The amount of water desorbed at time t $\left(M_{H_{2}O}\left(t\right)\right)$ was calculated according to the following equation:

(2) $$M_{H_{2}O}\left(t\right)=\;C_{0}\;-\;C\left(t\right)$$

where $C_{0}$ is the initial sample water content. The amount of water desorbed at equilibrium $\left(M_{H_{2}O}^{\infty }\right)$ was estimated according to the following expression:

(3) $$M_{H_{2}O}^{\infty }=\;C_{0}\;-\;C_{\infty }$$

where $C_{\infty }$ is the sample water content at the end of the dehydration.

### 2.5. Energy Consumption of the By-Product’s Dehydration Process

The procedure used by Le Rose et al. [19] to determine the amount of energy consumed per gram of dehydrating by-product ($\tilde{E}\left(t\right)$) was used in this work. In summary, the by-product dehydration kinetic was described using the following relationships:

$$0\;\leq \;t\;\leq \;t_{c}$$

(4) $$M_{H_{2}O}\left(t\right)=k_{1}\cdot t$$

$$t>t_{c}$$

(5) $$M_{H_{2}O}\left(t\right)=k_{1}\cdot t_{c}+K_{2}\cdot \left\{1-exp\left[-\left(t-t_{c}\right)\cdot \left(\frac{k_{1}}{k_{2}}\right)\right]\right\}$$

where $k_{1}$ is the desorption rate during the 1st Stage $\left[\frac{g\;desorbed\;water}{100\;g\;dry\;matter}\cdot \frac{1}{min}\right]$; $k_{2}$ is the maximum amount of water desorbed during the 2nd Stage $\left[\frac{g\;desorbed\;water}{100\;g\;dry\;matter}\right]$; $t_{c}$ is the moment in which the transition from one stage to another takes place; and t is the dehydration time. The reader is invited to refer to the study of Conte et al. [20] for a detailed description of the model and of the hypotheses used to derive it.

As reported in a previous paper [21], the time needed to reach a given extent of dehydration ($t_{ext\%}$) is related to the extent of dehydration (ext%) through the following expressions:

$$0\;\leq \;ext\%\;\leq \;\frac{k_{1}\cdot t_{c}}{M_{H_{2}O}^{\infty }}\cdot 100$$

(6) $$t_{ext\%}=\frac{M_{H_{2}O}^{\infty }}{k_{1}\cdot 100}\cdot ext\%$$

$$ext\%>\frac{k_{1}\cdot t_{c}}{M_{H_{2}O}^{\infty }}\cdot 100$$

(7) $$t_{ext\%}=t_{c}-ln\left(\frac{k_{1}\cdot t_{c}+k_{2}-ext\%\cdot \frac{M_{H_{2}O}^{\infty }}{100}}{k_{2}}\right)\cdot \frac{k_{2}}{k_{1}}$$

The amount of energy consumed per gram of dehydrating by-product is related to the extent of the dehydration process through the following expressions:

$$0\;\leq \;ext\%\;\leq \;\frac{k_{1}\cdot t_{c}}{M_{H_{2}O}^{\infty }}\cdot 100$$

(8) $$\tilde{E}\left(ext\%\right)=\frac{\gamma \cdot \left(\frac{M_{H_{2}O}^{\infty }}{k_{1}\cdot 100}\cdot ext\%\right)}{m_{Tot}^{0}\cdot \left(1-\frac{x_{dm}^{0}}{100}\cdot ext\%\cdot \frac{M_{H_{2}O}^{\infty }}{100}\right)}$$

$$ext\%>\frac{k_{1}\cdot t_{c}}{M_{H_{2}O}^{\infty }}\cdot 100$$

(9) $$\tilde{E}\left(ext\%\right)=\frac{\gamma \cdot \left[t_{c}-ln\left(\frac{k_{1}\cdot t_{c}+k_{2}-ext\%\cdot \frac{M_{H_{2}O}^{\infty }}{100}}{K_{2}}\right)\cdot \frac{k_{2}}{k_{1}}\right]}{m_{Tot}^{0}\cdot \left(1-\frac{x_{dm}^{0}}{100}\cdot ext\%\cdot \frac{M_{H_{2}O}^{\infty }}{100}\right)}$$

where γ is the energy power per unit of time provided to the dehydrator; $m_{Tot}^{0}$ is the initial value of the mass of dehydrating by-product; and $x_{dm}^{0}$ is the initial value of dry matter mass fraction. The reader is invited to refer to the study of Le Rose et al. [19] for a detailed description of the procedure used to determine the amount of energy consumed per gram of dehydrating by-product.

### 2.6. Process Variables, Sample Size and Rationale of Experimental Design

The process variables investigated in the present study were the dehydration temperature, the freezing–thaw pretreatment, and the cold-press extraction process. The factors that directly affect the dehydration process, and consequently the associated energy consumption, are the structural characteristics of the by-product and its initial moisture content. These characteristics are, in turn, modified by the pretreatments applied before dehydration. For this reason, the present study did not investigate the effect of dehydration temperature on the freezing–thaw pretreatment or on the cold-press dewatering, as such effects would have no physical or methodological basis within the experimental design. Therefore, energy for the frozen and unfrozen whole tomato by-products without any cold-press dewatering step was assessed only at 60 °C.

Each processing condition (fresh or frozen material, with or without cold-press extraction, and each drying temperature) was carried out as an independent processing batch. Process replication was not performed because of the large amount of raw material required at each drying temperature (about 65 kg). The workflow to apply the cold-press extraction requested a substantial quantity of by-products: about 30 kg for fresh raw material and about 35 kg for frozen raw material, for the subsequent drip loss before processing. For drying at 60 °C, additional by-product amounts were used: 6 kg of fresh by-products and approximately 10 kg of frozen tomato by-products.

## 3. Results and Discussion

### 3.1. Dehydration Kinetics

**Figure 1:**
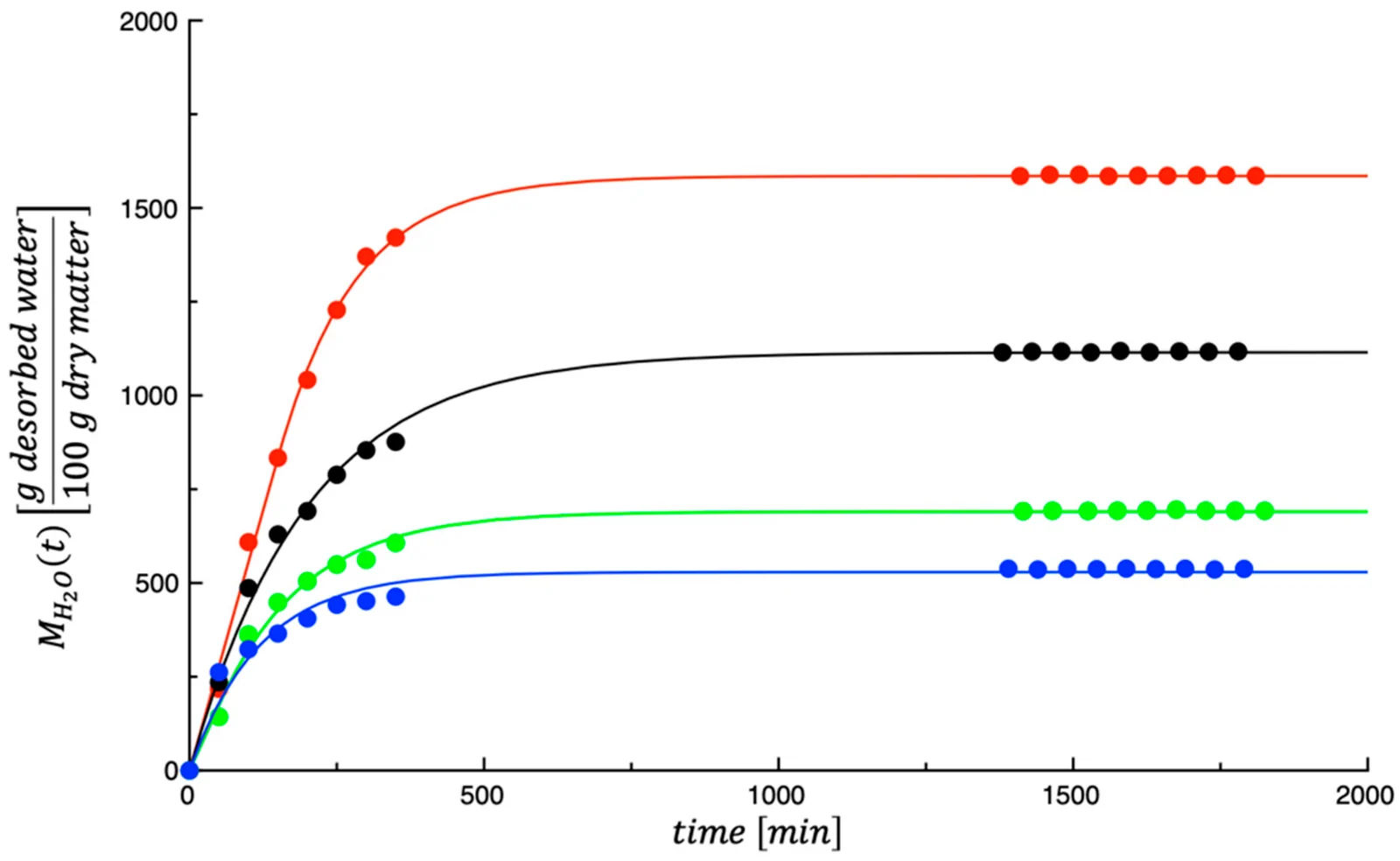
Dehydration kinetics at 60 °C; 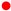 Unfrozen whole tomato. 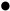 Thawed whole tomato; 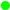 Unfrozen whole tomato + Extractor; 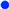 Thawed whole tomato + Extractor.

**Figure 2:**
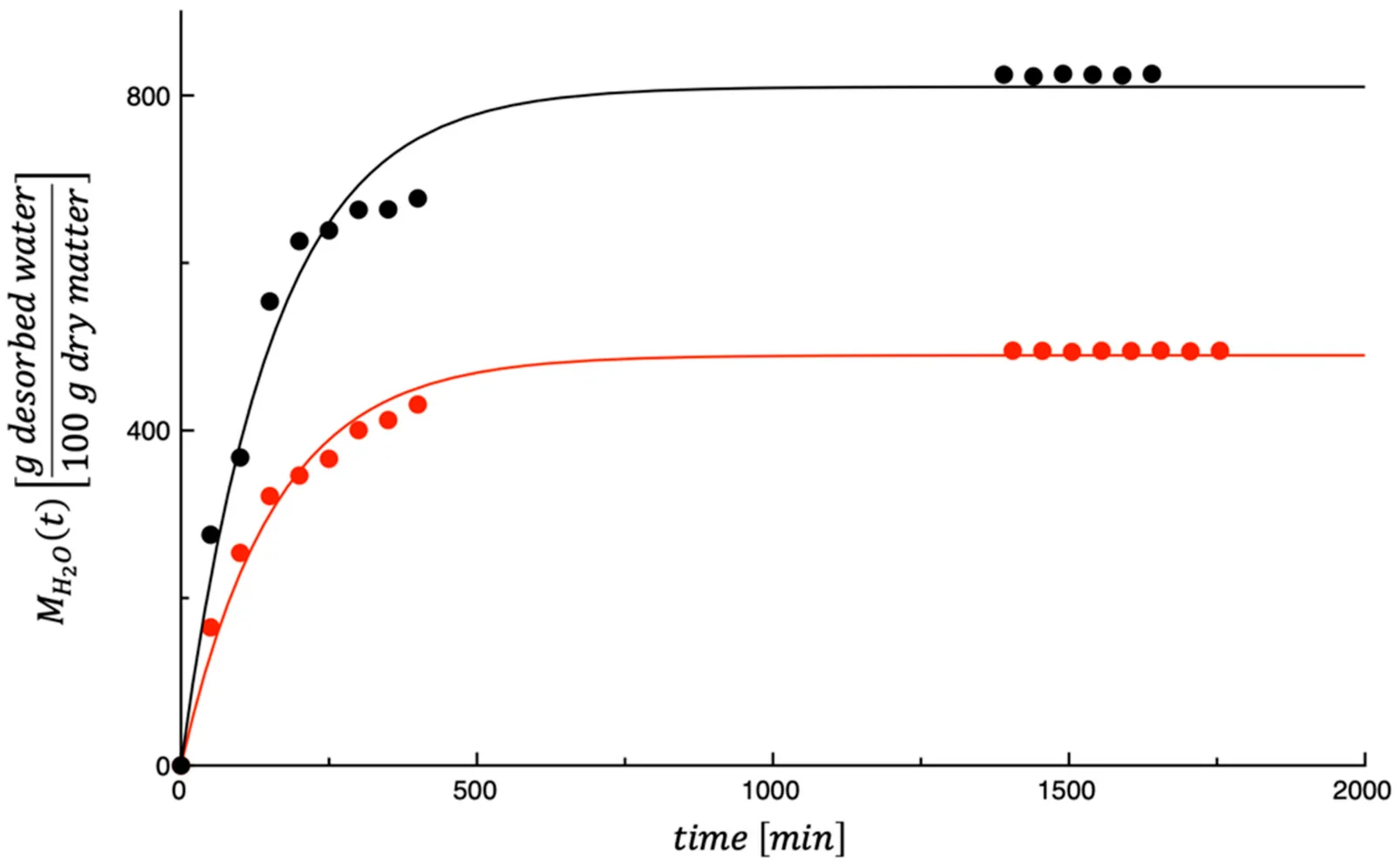
Dehydration kinetic at 50 °C; 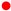 Thawed whole tomato + Extractor. 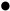 Unfrozen whole tomato + Extractor.

**Figure 3:**
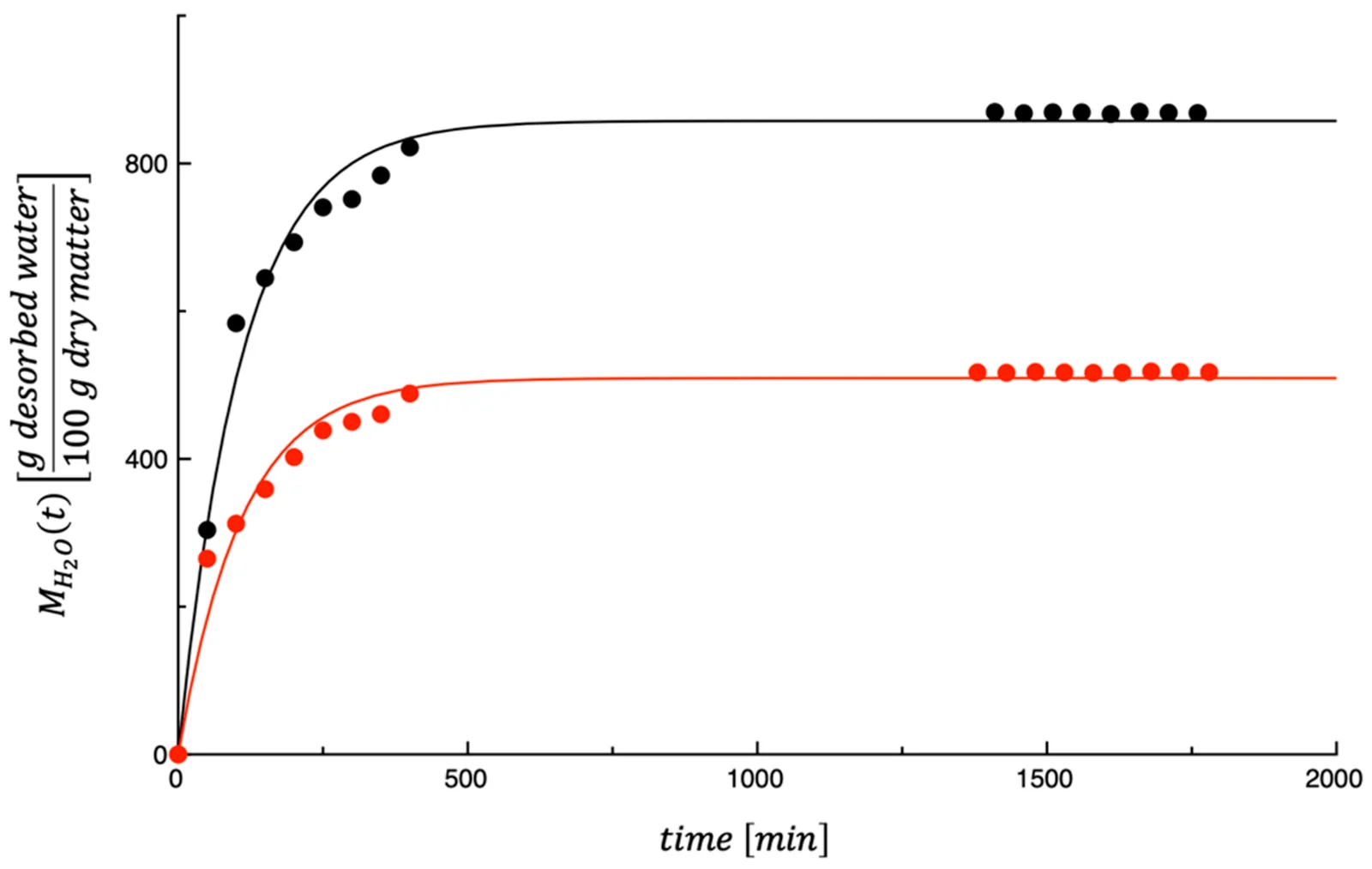
Dehydration kinetic at 70 °C; 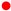 Thawed whole tomato + Extractor. 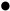 Unfrozen whole tomato + Extractor.

Figure 1, Figure 2 and Figure 3 show the dehydration kinetics of the investigated by-product at different temperatures. The four data sets shown in Figure 1 correspond to the four pretreatments applied to the samples prior to the dehydration step at 60 °C, namely: Unfrozen whole tomato, Thawed whole tomato, Unfrozen whole tomato + Extractor, and Thawed whole tomato + Extractor. By contrast, Figure 2 and Figure 3 report only the data for Thawed whole tomato + Extractor and Unfrozen whole tomato + Extractor at 50 and 70 °C, respectively. The curves shown in the figures are the best fit of the model proposed by Conte et al. [20] (i.e., Equations (4) and (5)) to the dehydration kinetic data. The values of the model’s parameter obtained by fitting the dehydration kinetics are listed in Table 1, Table 2 and Table 3. The γ values shown in Table 1, Table 2 and Table 3 were obtained by fitting the dehydrator’s energy consumption data with a straight line passing through the origin.

The mean relative deviation modulus $\left(\bar{E}\%\right)$ was used to measure the goodness of fit, according to a similar approach also used in the work of Lordi et al. [18]:

(10) $$\bar{E}\%\;=\;\frac{100}{N}\cdot \sum_{i=1}^{i=N}\frac{\left|M_{i}^{exp}-\;M_{i}^{pred}\right|}{M_{i}^{exp}}$$

where N is the number of experimental data; $M_{i}^{exp}$ is the experimental value; and $M_{i}^{pred}$ is the predicted value. As can be deduced from the $\bar{E}\%$ values shown in Table 1, Table 2 and Table 3, the model proposed by Conte et al. [20] adequately describes the dehydration kinetic of the by-products studied in this work. Regarding the fitting of the dehydrator’s energy consumption data (i.e., those used to obtain the γ values), the $\bar{E}\%$ values are in some cases slightly high, most probably due to the simplicity of the model used for the fitting (i.e., a line passing through the origin).

According to the literature, food dehydration proceeds through a sequence of distinct drying stages. The model proposed by Conte et al. [20] assumes that drying proceeds through two distinct stages. During the initial stage (1st stage), vaporization occurs at the surface of the by-product, where free water is still available. After this stage, the dehydration rate progressively decreases until it reaches zero (2nd stage). The time at which the transition between the two stages occurs is denoted as $t_{c}$. As shown by the data reported in Table 1, Table 2 and Table 3, the different pre-treatments applied and the various dehydration temperatures influenced the mechanism. In fact, in most cases the value of $t_{c}$ is so small that the 1st stage can be considered absent. Only in the two cases of Unfrozen whole tomato and Unfrozen whole tomato + Extractor was a sufficiently high $t_{c}$ value observed, thus suggesting the presence of the 1st Stage. In the case of the Unfrozen whole tomato, the presence of the 1st Stage is associated with the high water content of the by-product. In contrast, for the Unfrozen whole tomato + Extractor sample, the occurrence of the 1st Stage, although to a lesser extent than in the previous sample, can be attributed to the fact that the extraction step was not sufficiently effective in removing water. In other case studies also dealing with by-products, we observed a high $t_{c}$. For example, when peels and seeds from tomatoes were dehydrated, $t_{c}$ values ranged from about 62 to 170 min depending on the temperature [22]. In the case of peels from prickly pears, Panza et al. [21] also observed a $t_{c}$ value around 250 min. In both cases the authors observed that the value of $t_{c}$ decreased when increasing the dehydration temperature from 50 to 70 °C because they assumed that during the 1st stage, the water evaporation rate from the by-product surface is the main factor limiting the dehydration rate. This aspect, in turn, depends on the enthalpy of the vaporization and on the rate with which the energy is transferred to the food.

Data reported in Table 1, Table 2 and Table 3 (i.e., $t_{c}$, $k_{1}$, and $k_{2}$) were used to predict the values of $t_{99\%}$ and $M_{H_{2}O}\left(t_{99\%}\right)$, also reported in the same tables. The former was predicted using Equations (6) and (7), while the latter was predicted using Equations (4) and (5). As shown by the data reported in the tables, the cold-press dewatering reduces the amount of water removed during the subsequent dehydration step (i.e., $M_{H_{2}O}\left(t_{99\%}\right)$). This effect is observed both when the by-product has been previously thawed and when it has not undergone freezing. The result is exactly in line with expectations and confirms that the cold-press dewatering effectively reduces the amount of water that must be removed during the dehydration phase. Data reported in the tables also highlight the effect of freezing on $M_{H_{2}O}\left(t_{99\%}\right)$. The results indicate that freezing decreases the quantity of water to be eliminated during dehydration. This behaviour was observed at all three temperatures examined (i.e., 50, 60, and 70 °C). This effect is most likely attributable to the disruption of the tomato’s cellular structure caused by freezing [23], which enhances the efficiency of the dewatering process. Data in Table 1, Table 2 and Table 3 also show that freezing reduces the amount of water removed during dehydration even when the extractor is not used. This outcome is again related to the cellular structure breakdown induced by freeze–thaw pretreatment. Freezing causes the formation of ice crystals inside plant tissues. These crystals disrupt cell walls and membranes, increasing tissue porosity. After thawing, this structural damage facilitates the release of intracellular water, making it more readily available for removal during dehydration. As a result, water diffusion from the interior of the vegetable to the surface is enhanced, which can shorten drying time. Second, during freezing, a portion of the water is converted into ice and segregated from soluble solids. Upon thawing, some of this water can be lost as drip loss (exudate), effectively reducing the initial moisture content of the vegetable before dehydration even begins [15,24]. This means that the total amount of water that the drying process needs to remove is lower. Other findings in the literature demonstrated that the increased permeability of tissues after freeze–thaw treatment lowers the resistance to mass transfer. This improves drying efficiency, allowing dehydration to proceed at lower temperatures or in shorter time, which can also help preserve heat-sensitive nutrients and bioactive compounds [16,25]. Furthermore, the rupture of the cell wall caused by ice crystals allows intracellular water to become free water. This reduces the compressive strength of the tissue, improving the release of liquid during mechanical pressing, thus increasing yield [26].

**Figure 4:**
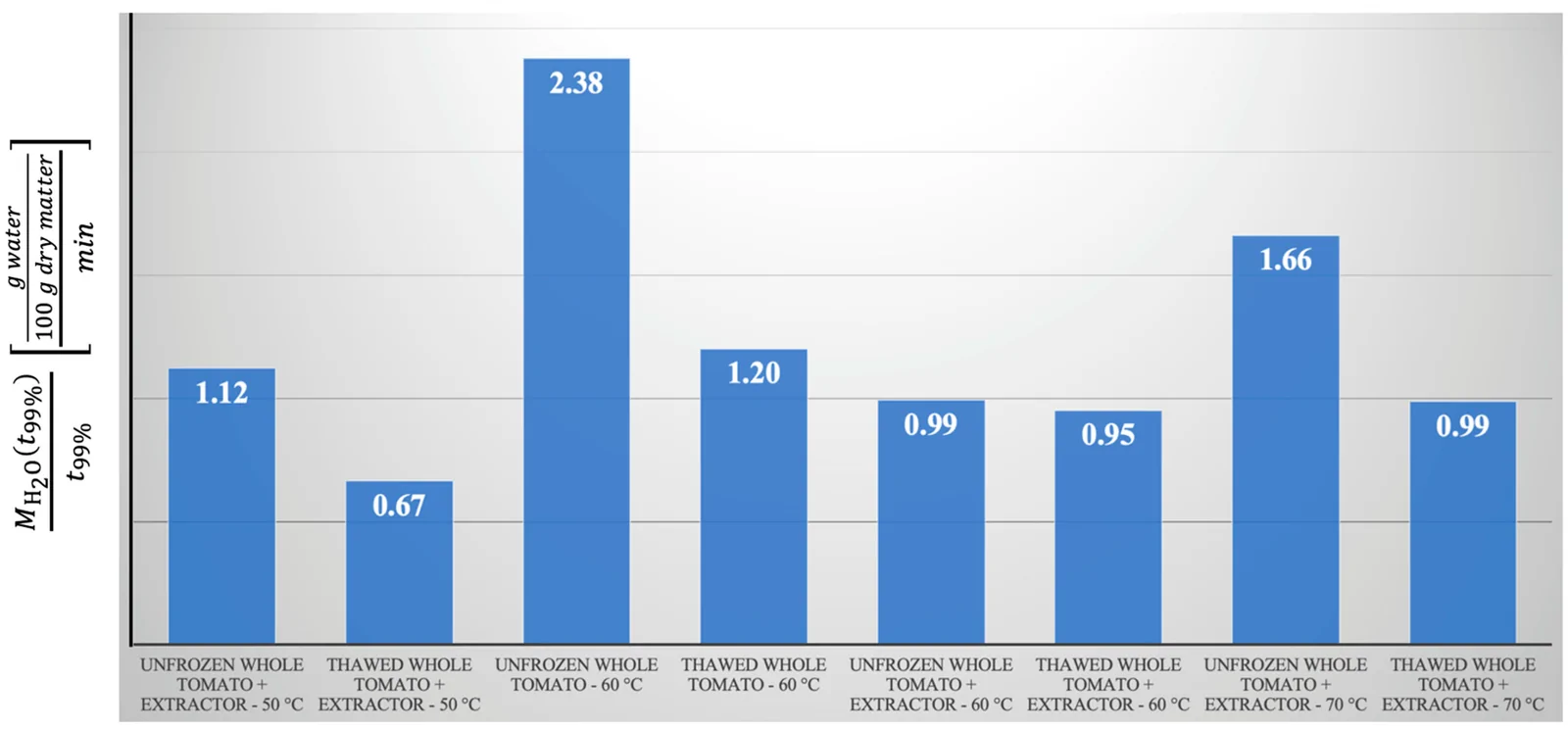
Average dehydration rate $\frac{M_{H_{2}O}\left(t_{99\%}\right)}{t_{99\%}}$ $\left[\frac{\frac{g\;water}{100\;g\;dry\;matter}}{\min}\right]$ for unfrozen and thawed whole tomato, with and without cold-press dewatering, dehydrated at 50 °C, 60 °C and 70 °C.

Regarding $t_{99\%}$, data reported in Table 1, Table 2 and Table 3 suggest that this value is not strictly correlated with the amount of water to be removed during dehydration. This is because freezing alters the cellular structure of the tomato, thereby modifying the water diffusion mechanism through the by-product during the dehydration process. Figure 4 reports the average dehydration rate $\frac{M_{H_{2}O}\left(t_{99\%}\right)}{t_{99\%}}$ for all the samples examined. Data shown in this figure indicate that freezing reduces the average water removal rate both in the presence and in the absence of the cold-press dewatering process. When this step is included, the slower water removal observed after freezing and dewatering may be associated with structural modifications that could influence mass transfer during drying. Dedicated structural analyses, such as those measuring porosity, bulk density, or microstructural characterization, would be required to confirm this hypothesis. Even when cold-press dewatering is not applied, a reduction in the average dehydration rate is still observed because of freezing. This effect is most likely due to the release of sugars from the cells, during which dehydration forms a barrier to water migration (indeed, a whitish layer covering the by-product was observed after removing the by-product from the dryer). A similar phenomenon was also observed by Ratti [27]; especially in products with a high sugar content, when water migrates to the surface, it carries the solutes with it and once the water evaporates, the sugars settle and crystallize, making the surface impermeable. The study conducted by Lewicki [28] explicitly explains how pretreatments that cause tissue disintegration (such as freezing) lead to the formation of a surface film of solutes, creating a dense structure that prevents water evaporation.

### 3.2. Energy Consumption

**Figure 5:**
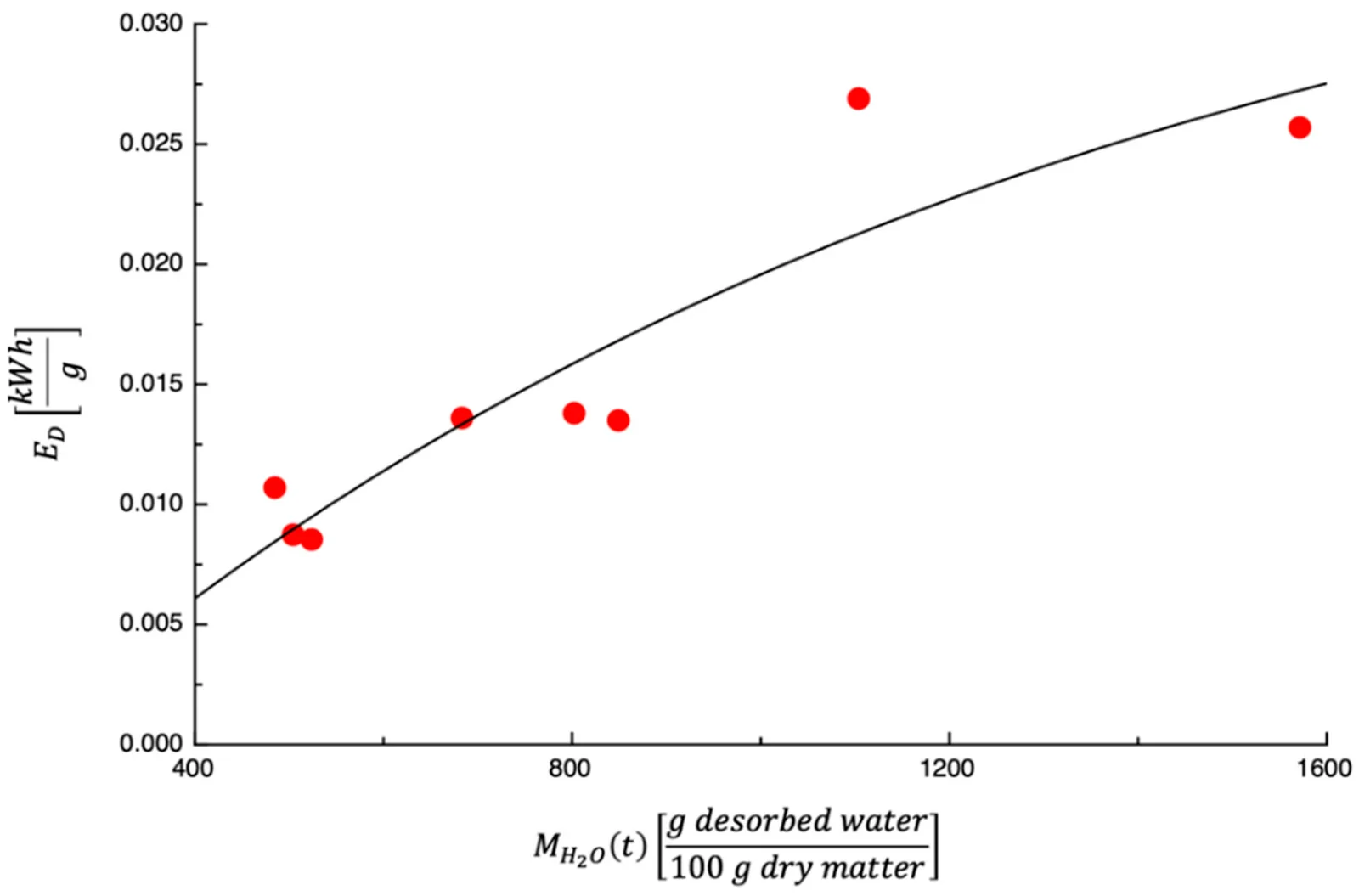
Values of energy consumption for dehydration ($E_{D}$) as a function of $M_{H_{2}O}(t_{99\%})$.

Table 4 reports the energy consumption associated with the three stages of the process used to convert the by-product into powder (i.e., dewatering, dehydration, and grinding). It is important to note that all energy data are expressed per gram of final powder obtained from grinder. Regarding the energy consumed by the dehydrator (i.e., $E_{D}$), this value was calculated using $\tilde{E}\left(99\%\right)$ and expressing it per gram of powder at the grinder exit, rather than per gram of product leaving the dehydrator. As shown by the data in the table, there is a substantial difference in the energy required by the three stages of the powder production process. In particular, the dehydration is by far the most energy-intensive step. This is due to the involvement of the phase change of water from liquid to vapor during this stage. As further evidence of the observations discussed above, Figure 5 shows $E_{D}$ as a function of $M_{H_{2}O}(t_{99\%})$. The figure includes data for all the eight samples examined in this study. The curve shown in the figure is intended solely to highlight the trend of data and it is not representative of any underlying model. As can be observed from data shown in the figure, there is a clear trend in which $E_{D}$ increases with increasing $M_{H_{2}O}(t_{99\%})$. The scatter in the data suggests that the amount of evaporated water is not the sole factor influencing the energy consumption of the dehydrator because the temperature and the average dehydration rate are certainly relevant. This result is consistent with what was reported by Urbano et al. [29]. In fact, these Authors demonstrated that both the drying temperature and initial product moisture content of agricultural products influence drying performance and consequently the energy consumption. Specifically, as the drying temperature increased, the rate of water evaporation increased.

However, as shown in Figure 5, the amount of evaporated water is by far the most important factor. As reported beforehand, this is attributable to the large amount of energy required for evaporation, due to the latent heat of vaporization. Bayana et al. [10] also carried out a study on the drying of tomato pomace, demonstrating that the energy demand for dehydrating the by-products is mainly influenced by the energy used for the evaporation. The energy consumption of the dehydration phase is also supported by Akinbi et al. [30], who analysed the drying characteristics of tomato slices. Their thermodynamic analysis demonstrated that the energy required for water vaporization is the main factor regulating the intensity of the dehydration process.

**Figure 6:**
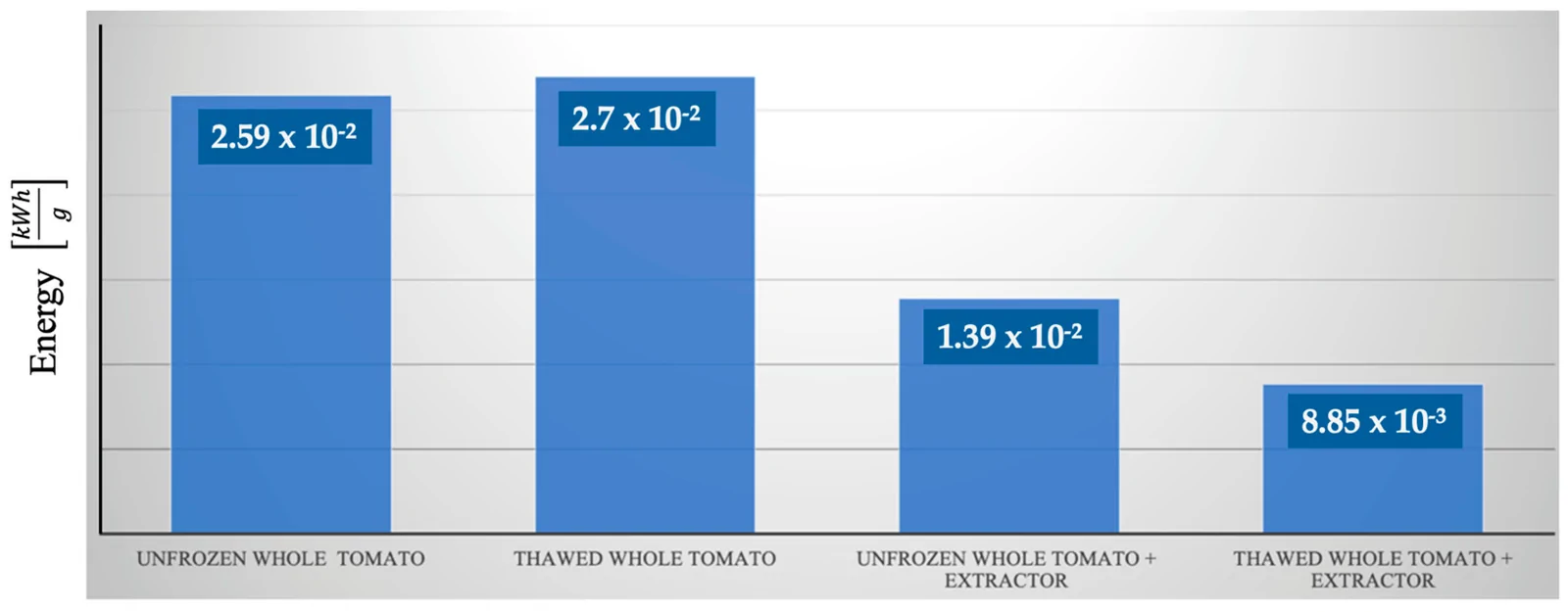
Total energy required to produce the dehydrated by-product powder at 60 °C.

Figure 6 shows the energy required to dehydrate tomato by-products at 60 °C. As before, the reported energy values are expressed per unit mass at the grinder exit. It is worth noting that this energy consumption was associated with the energy specifically required for the cold-press dewatering, dehydration and grinding process. As evidenced by the data in the figure, there is a substantial difference among the samples dehydrated at 60 °C. The presence of the cold-press dewatering step results in a considerable energy saving, regardless of whether the by-product was previously frozen or not. This is because the extraction step reduces the amount of water that must be removed during dehydration (see Table 1, Table 2 and Table 3). Consequently, this step allows a reduction in the energy associated with the phase change of water. The substantial energy saving provided by the extraction step is consistent with the general principle that cold-press dewatering is more energy efficient than thermal drying, as the energy required to remove liquid water is orders of magnitude lower than the latent heat of vaporization; the latent heat of vaporization that must be supplied for moisture removal makes thermal drying likely the most energy-intensive industrial process [31].

Regarding the freezing of the by-product, data show that the disruption of the cellular structure enhances the efficiency of the dewatering step and consequently reduces the amount of water that must be removed during dehydration, which in turn leads to a significant energy saving. The observation that the freezing step enhances extraction efficiency by disrupting the cellular matrix aligns with findings presented by Ershidat [32], who demonstrated that the ‘Blending the Frozen’ method increases dewatering efficiency from tomato pomace due to the mechanical disruption of cell walls caused by ice crystal formation. For whole tomato, as previously discussed, freezing decreases the average dehydration rate. This evidence could be associated with the formation of a sugar-rich barrier that hinders water removal. Dedicated microstructural and compositional analyses would be necessary to verify this mechanism in tomato by-products. However, in terms of energy demand, the difference between the two whole-tomato samples is minimal. Considering all the samples dehydrated at 60 °C, the Thawed whole tomato + Extractor configuration provides the greatest energy savings. Using any of the other processing methods investigated would lead to an increase in energy consumption. It is worth noting that depending on the difference between the rate at which the by-product is generated and the rate at which it can be processed, the freezing step may or may not be required for by-product stabilization. Therefore, according to production needs, to assess the influence of cold-press dewatering on the energy, the relevant energy consumption comparisons are those between Thawed whole tomato and Thawed whole tomato + Extractor when freezing is necessary, and between Unfrozen whole tomato and Unfrozen whole tomato + Extractor when freezing is not required. In the first case, not using the extractor results in a 205% increase in energy consumption, whereas in the second case it leads to an 86% increase.

**Figure 7:**
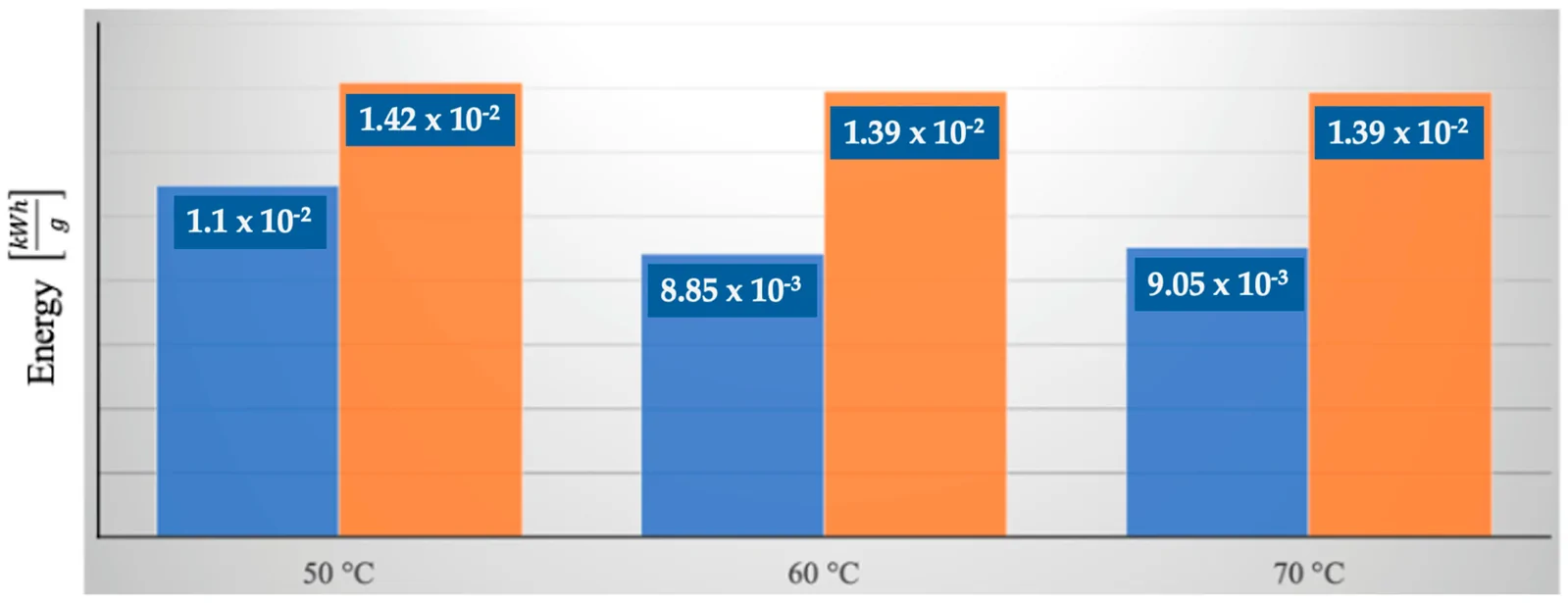
Values of energy consumption associated to cold-press dewatering, dehydration and grinding, at the three temperatures investigated (50 °C, 60 °C and 70 °C). Blue = thawed whole tomato; Orange = unfrozen whole tomato.

To assess the effect of dehydration temperature on energy consumption, Figure 7 reports the energy consumption values associated with cold-press dewatering, dehydration and grinding, at the three temperatures investigated. Data shown in the figure refer only to the samples processed by cold-press dewatering, as the results at 60 °C clearly demonstrate that the use of this step leads to a substantial energy saving. Data shown in the figure confirm the observations previously discussed, namely that freezing alters the structure of the by-product, increases the efficiency of the dewatering step, and consequently reduces the energy demand of the dehydrator. Indeed, at both 50 °C and 70 °C, a considerable reduction in energy consumption is observed when the thawed by-product is used. The data presented in Figure 7 also show that the effect of dehydration temperature, if present, is very small. In fact, only a slight reduction in the energy demand is observed as the temperature increases.

## 4. Conclusions

The study applied to by-products made up of whole tomatoes demonstrated the effects of a freezing–thaw pretreatment applied prior to dehydration, with and without the application of a further cold-press dewatering process before drying. Three different dehydration temperatures were tested, namely 50, 60 and 70 °C. The results show that the cold-press dewatering reduced the amount of water removed during the subsequent dehydration step. As regards freezing and thawing, it can further reduce the water load to be removed both by promoting partial water loss during thawing and by enhancing internal water mobility. These effects translate into a faster dehydration process. Considering that the dehydration is by far the most energy-intensive step, thawed whole tomato and use of cold-press dewatering before dehydration provided the greatest energy savings. As regards the effect of dehydration temperature on energy consumption, the results confirm the observation still reported regarding freezing, i.e., its ability to alter the structure of the by-product and increase the efficiency of the mechanical extractor, with a consequent reduction in the energy demand of the dehydrator. Indeed, at each tested temperature, a considerable reduction in energy consumption is observed when the thawed by-product from the extractor is used. Among the three temperatures, small variations were found, and only a slight energy reduction was observed as the temperature increased. Future research should investigate the combined techno-economic and environmental performance of these pretreatments at industrial scale, while also evaluating their effects on the recovery and preservation of high-value compounds, thereby supporting the implementation of sustainable tomato by-product valorisation pathways and enabling us to draw broader conclusions regarding the overall sustainability of the process. In addition, freezing parameters such as the freezing rate, temperature evolution at the sample core, and drip loss could be also monitored as variables influencing the microstructure of the tissue and the subsequent extraction and drying behaviour.

## Figures and Tables

**Table 1:** Values of model’s parameter obtained by fitting the dehydration kinetics along with the calculated values of $\bar{E}\%$ for drying at 50 °C.

	50 °C	
	Unfrozen Whole Tomato + Extractor	Thawed Whole Tomato + Extractor
$t_{c}\;\left[\min\right]$	5.97·10^−6^	1.49·10^−6^
$k_{1}\left[\frac{g\;desorbed\;water}{100\;g\;dry\;matter}\cdot \frac{1}{\min}\right]$	5.22	3.10
$k_{2}\;\left[\frac{g\;desorbed\;water}{100\;g\;dry\;matter}\right]$	810.26	489.71
$\gamma \;\left[\frac{\mathrm{kWh}}{\min}\right]$	1.49·10^−2^	1.48·10^−2^
$\bar{E}\%$ Dehydration Kinetic	5.39	4.14
$\bar{E}\%$ Energy Consumption	1.69	5.46
$t_{99\%}\;\left[\min\right]$	715.29	728.15
$M_{H_{2}O}\left(t_{99\%}\right)$ $\left[\frac{g\;water}{100\;g\;dry\;matter}\right]$	802.16	484.82

**Table 2:** Values of model’s parameter obtained by fitting the dehydration kinetics along with the calculated values of $\bar{E}\%$ for drying at 60 °C.

	60 °C			
	Unfrozen Whole Tomato	Thawed Whole Tomato	Unfrozen Whole Tomato + Extractor	Thawed Whole Tomato + Extractor
$t_{c}\;\left[\min\right]$	1.53·10^2^	3.06·10^−2^	50.93	1.42·10^−5^
$k_{1}\left[\frac{g\;desorbed\;water}{100\;g\;dry\;matter}\cdot \frac{1}{\min}\right]$	5.56	5.59	3.47	4.43
$k_{2}\;\left[\frac{g\;desorbed\;water}{100\;g\;dry\;matter}\right]$	734.85	1114.69	513.31	529.02
$\gamma \;\left[\frac{\mathrm{kWh}}{\min}\right]$	1.72·10^−2^	1.74·10^−2^	1.72·10^−2^	1.71·10^−2^
$\bar{E}\%$ Dehydration Kinetic	2.64	1.75	3.18	5.14
$\bar{E}\%$ Energy Consumption	8.78	8.86	7.04	4.01
$t_{99\%}\;\left[\min\right]$	660.24	918.83	687.6	550.42
$M_{H_{2}O}\left(t_{99\%}\right)$ $\left[\frac{g\;water}{100\;g\;dry\;matter}\right]$	1571.50	1103.71	683.30	523.72

**Table 3:** Values of model’s parameter obtained by fitting the dehydration kinetics along with the calculated values of $\bar{E}\%$ for drying at 70 °C.

	70 °C	
	Unfrozen Whole Tomato + Extractor	Thawed Whole Tomato + Extractor
$t_{c}\;\left[\min\right]$	3.13·10^−6^	1.62·10^−2^
$k_{1}\left[\frac{g\;desorbed\;water}{100\;g\;dry\;matter}\cdot \frac{1}{\min}\right]$	7.73	4.60
$k_{2}\;\left[\frac{g\;desorbed\;water}{100\;g\;dry\;matter}\right]$	857.64	509.29
$\gamma \;\left[\frac{\mathrm{kWh}}{\min}\right]$	2.00·10^−2^	1.83·10^−2^
$\bar{E}\%$ Dehydration Kinetic	2.90	4.40
$\bar{E}\%$ Energy Consumption	11.01	14.46
$t_{99\%}\;\left[\min\right]$	511.01	510.35
$M_{H_{2}O}\left(t_{99\%}\right)$ $\left[\frac{g\;water}{100\;g\;dry\;matter}\right]$	849.06	504.27

**Table 4:** $E_{D}$ is the energy consumed by the dehydrator per gram of product exiting the grinder; $E_{E}$ is the energy consumed by the cold-press dewatering step per gram of product exiting the grinder; $E_{G}$ is the energy required for grinding per gram of product exiting the grinder.

Energy	50 °C		60 °C				70 °C	
	Unfrozen Whole Tomato + Extractor	Thawed Whole Tomato + Extractor	Unfrozen Whole Tomato	Thawed Whole Tomato	Unfrozen Whole Tomato + Extractor	Thawed Whole Tomato + Extractor	Unfrozen Whole Tomato + Extractor	Thawed Whole Tomato + Extractor
$E_{D}$ $\left[\frac{\mathrm{kWh}}{g}\right]$	1.38·10^−2^	1.07·10^−2^	2.57·10^−2^	2.69·10^−2^	1.36·10^−2^	8.54·10^−3^	1.35·10^−2^	8.74·10^−3^
$E_{E}$ $\left[\frac{\mathrm{kWh}}{g}\right]$	3.49·10^−4^	2.32·10^−4^	_	_	2.78·10^−4^	2.65·10^−4^	2.99·10^−4^	2.61·10^−4^
$E_{G}$ $\left[\frac{\mathrm{kWh}}{g}\right]$	4.98·10^−5^	4.64·10^−5^	1.28·10^−4^	5.52·10^−5^	4.02·10^−5^	4.61·10^−5^	5.75·10^−5^	4.12·10^−5^

## Data Availability

The original contributions presented in the study are included in the article, and further inquiries can be directed to the corresponding author.
